# Loneliness, spiritual well-being, and death perception, as well as their risk factors in urological cancer patients

**DOI:** 10.1590/1414-431X2023e12915

**Published:** 2023-08-14

**Authors:** Chunmei Xia, Xu Zhao, Boyi Li, Bingjie Qi, Yujia Hong

**Affiliations:** 1 Department of Urology, The First Affiliated Hospital of Harbin Medical University, Harbin, China; 2 Department of Neurosurgery, The First Affiliated Hospital of Harbin Medical University, Harbin, China

**Keywords:** Urological cancer, Loneliness, Spiritual well-being, Death perception, Risk factors

## Abstract

Cancer patients commonly suffer from loneliness, poor spiritual status, and fear of death; however, these evaluations are rarely revealed in urological cancer patients. Thus, this study aimed to assess the loneliness, spiritual well-being, and death perception, as well as their risk factors in urological cancer patients. A total of 324 urological (including renal, bladder, and prostate) cancer patients and 100 healthy controls were included. The University of California and Los Angeles loneliness scale (UCLA-LS), functional assessment of chronic illness therapy-spiritual well-being (FACIT-Sp), and death attitude profile-revised (DAP-R) scores were evaluated. The results showed that the UCLA-LS score was higher, but the FACIT-Sp score was lower in urological cancer patients than in healthy controls. According to the DAP-R score, fear of death, death avoidance, and approaching death acceptance were elevated, but neutral acceptance was lower in urological cancer patients than in healthy controls. Among urological cancer patients, the UCLA-LS score was highest but the FACIT-Sp score was lowest in bladder cancer patients; regarding the DAP-R score, fear of death and death avoidance were highest, but approaching death acceptance was lowest in bladder cancer patients. Interestingly, single/divorced/widowed status, bladder cancer diagnosis, higher pathological grade, surgery, systemic treatment, and local treatment were independent factors for higher UCLA-LS score or lower FACIT-Sp score. In conclusion, urological cancer (especially bladder cancer) patients bear increased loneliness and reduced spiritual well-being; they also carry higher fear of death, death avoidance, and approaching death acceptance but lower neutral acceptance of death.

## Introduction

Urological cancers affect the organs and structures of the male and female urinary systems, as well as the male reproductive system (1). Prostate, bladder, and renal cancers are the three main types of urological cancer, which account for approximately 3.8, 2.1, and 1.8% of all cancer-related mortalities worldwide according to the Global Cancer Statistics 2020 (2). Benefitting from the advancement of treatment strategies, the prognosis of urological cancer patients has been improved to some extent (3-5). Unfortunately, urological cancers and the corresponding treatments unavoidably affect patients' physical and psychological status (6-8). Urological cancer patients usually suffer from negative emotions, such as loneliness, unstable spirituality, and fear of death, which might ultimately influence their quality of life, treatment outcome, and even survival (9-11). Therefore, a better understanding of loneliness, spiritual status, and death perception, as well as their risk factors in urological cancer patients is crucial to improve the management of these patients. Several studies have investigated loneliness, spiritual well-being, and death perception in general cancer (such as lung cancer, breast cancer, brain cancer, colon cancer, etc.) patients, and have also explored the corresponding risk factors (12-14). For instance, one study illustrates that loneliness is generalized in cancer patients; in addition, family support and sharing of emotional stress are related to reduced loneliness in these patients (12). Married cancer patients have greater spiritual well-being compared to non-married patients (14). In addition, a previous study found that older age, male sex, widowed, and diagnosed with stage IV cancer (including breast, lung, gynecological, or gastrointestinal cancer) are correlated with death acceptance in cancer patients (15). This study intended to investigate loneliness, spiritual well-being, and death perception in urological cancer (including prostate, bladder, and renal cancers) patients, together with their risk factors and correlation.

## Material and Methods

### Subjects

A total of 324 urological cancer patients, including renal, bladder, and prostate cancers, were included consecutively between March 2021 and July 2022. The inclusion criteria involved: 1) diagnosed as urological cancer including renal, bladder, and prostate cancer; 2) older than 18 years; 3) willingness to participate; and 4) willingness and capable to fill the questionnaire by themselves. The exclusion criteria involved: 1) serious cognitive impairments or psychiatric disorders; 2) unable to understand the protocol; and 3) unable to communicate normally. Additionally, a total of 100 healthy subjects were recruited as healthy controls. The inclusion criteria involved: 1) no abnormalities in recent physical examinations; 2) matched age and gender to urological cancer patients; 3) older than 18 years; and 4) willing to participate and complete the questionnaire. The study had the approval of the Ethics Committee of the First Affiliated Hospital of Harbin Medical University (China). Each subject signed the informed consent.

### Data collection

The clinical characteristics of urological cancer patients were recorded, including: 1) demographics: age, gender, smoking history, drinking history, marital status, employment status, level of education, and location; and 2) disease history: hypertension, hyperlipidemia, and diabetes. Additionally, the disease features that were evaluated at diagnosis were also collected, including tumor type, pathological grade, and tumor-node-metastasis (TNM) stage. Pathological grade was assessed based on the following criteria: grade 1, well differentiation for renal and bladder cancer patients, or Gleason ≤6 for prostate cancer patients; grade 2, moderate differentiation for renal and bladder cancer patients, or Gleason =7 for prostate cancer patients; grade 3, poor differentiation for renal and bladder cancer patients, or Gleason ≥8 for prostate cancer patients. Additionally, treatment information was obtained, and then classified as surgery treatment, systemic treatment, local treatment, and other treatments.

### Assessment

The University of California and Los Angeles loneliness scale (UCLA-LS) was adopted for loneliness evaluation. The scale contains 20 items and scores range from 20-80 (16). A higher UCLA-LS score indicates higher levels of loneliness. Functional assessment of chronic illness therapy - spiritual well-being (FACIT-Sp) was adopted for spiritual perception evaluation. It contains 12 items and scores range from 0-48 (17). A higher FACIT-Sp score indicates better spiritual status. Death attitude profile-revised (DAP-R) was adopted for death perception evaluation and contains 32 items in 5 dimensions (18). A higher total score or mean score of each dimension indicates a higher subject's agreement with the dimension.

### Statistics

Data were analyzed by SPSS v24.0 (IBM Corp., USA). Figures were constructed using GraphPad Prism v8.0 (GraphPad Software Inc., USA). Comparison analysis was done by *t*-test. Association analysis was conducted by Pearson's test. Factors related to UCLA-LS and FACIT-Sp were screened by linear regression analysis. Factors shown in the univariate linear regression model were included by backward method in the multivariate linear regression model. P<0.05 was considered significant.

## Results

### Clinical features of urological cancer patients

The enrolled urological cancer patients had a mean age of 63.0±9.8 years with 250 (77.2%) males and 74 (22.8%) females. Regarding marital status, 227 (70.1%) patients were married and 97 (29.9%) patients were single/divorced/widowed. A total of 137 (42.3%) patients were employed and 187 (57.7%) patients were unemployed. In addition, 49 (15.1%) patients had a primary school level or less, 172 (53.1%) patients had a middle or high school level, and 103 (31.8%) patients had an undergraduate or above level of education. Notably, there were 86 (26.5%) renal cancer patients, 113 (34.9%) bladder cancer patients, and 125 (38.6%) prostate cancer patients. Concurrently, 68 (21.0%), 185 (57.1%), and 71 (21.9%) patients had pathological grade 1, 2, and 3 at diagnosis, respectively. There were 124 (38.3%), 109 (33.6%), 73 (22.5%), and 18 (5.6%) patients at TNM stage I, II, III, and IV, respectively. Detailed information on urological cancer patients is exhibited in Table 1.

**Table 1:** Clinical characteristics.

Characteristics	Urological cancer patients (n=324)
Demographics	
Age (years), mean±SD	63.0±9.8
Gender, n (%)	
Male	250 (77.2)
Female	74 (22.8)
Smoking history, n (%)	131 (40.4)
Drinking history, n (%)	104 (32.1)
Marital status, n (%)	
Married	227 (70.1)
Single/divorced/widowed	97 (29.9)
Employment status, n (%)	
Employed	137 (42.3)
Unemployed	187 (57.7)
Level of education, n (%)	
Primary school or less	49 (15.1)
Middle or high school	172 (53.1)
Undergraduate or above	103 (31.8)
Location, n (%)	
Urban	274 (84.6)
Rural	50 (15.4)
Disease history	
Hypertension, n (%)	137 (42.3)
Hyperlipidemia, n (%)	77 (23.8)
Diabetes, n (%)	54 (16.7)
Disease features	
Tumor type, n (%)	
Renal cancer	86 (26.5)
Bladder cancer	113 (34.9)
Prostate cancer	125 (38.6)
Pathological grade at diagnosis, n (%)	
Grade 1	68 (21.0)
Grade 2	185 (57.1)
Grade 3	71 (21.9)
TNM stage at diagnosis, n (%)	
I	124 (38.3)
II	109 (33.6)
III	73 (22.5)
IV	18 (5.6)
Treatment	
Surgery treatment, n (%)	303 (93.5)
Systemic treatment, n (%)	157 (48.5)
Local treatment, n (%)	143 (44.1)
Other treatments, n (%)	94 (29.0)

SD: standard deviation; TNM: tumor-node-metastasis.

### Comparison of UCLA-LS, FACIT-Sp, and DAP-R scores between urological cancer patients and healthy controls

**Figure 1:**
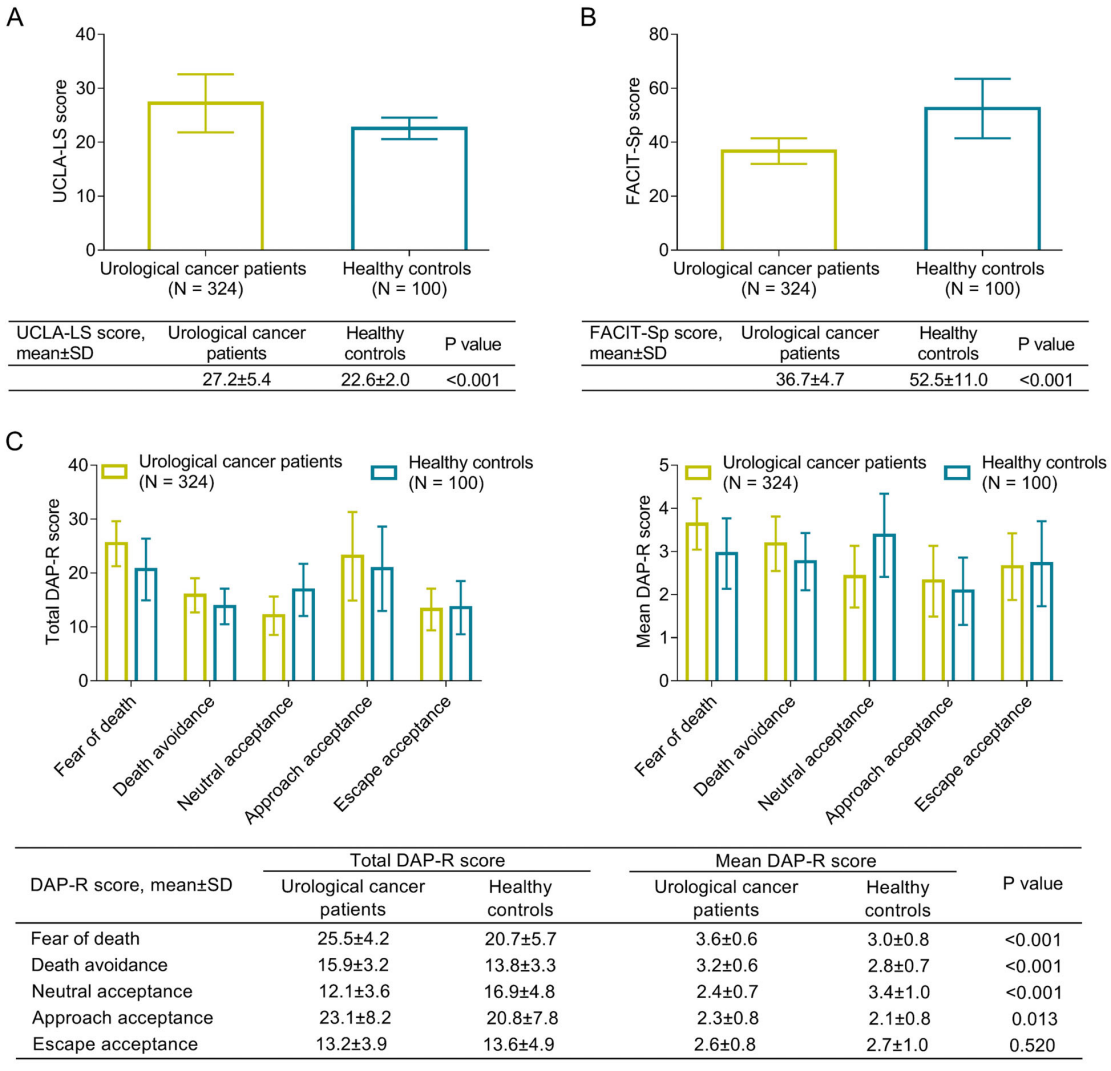
Comparison of University of California and Los Angeles loneliness scale (UCLA-LS) score (**A**), functional assessment of chronic illness therapy-spiritual well-being (FACIT-Sp) score (**B**), as well as total and mean death attitude profile-revised (DAP-R) score (**C**) between urological cancer patients and healthy controls. Data are reported as means±SD (*t*-test).

UCLA-LS score was higher in patients compared to controls (P<0.001) (Figure 1A). FACIT-Sp score was lower in patients than in controls (P<0.001) (Figure 1B). Regarding total DAP-R score, fear of death (P<0.001), death avoidance (P<0.001), and approaching death acceptance (P=0.013) were higher in patients compared to controls, whereas neutral acceptance was lower in patients compared to controls (P<0.001). The escape acceptance remained unchanged between patients and controls (P=0.520). Similar findings were found when comparing the mean DAP-R score between patients and controls (Figure 1C).

### Comparison of UCLA-LS, FACIT-Sp, and DAP-R scores among renal, bladder, and prostate cancer patients

**Figure 2:**
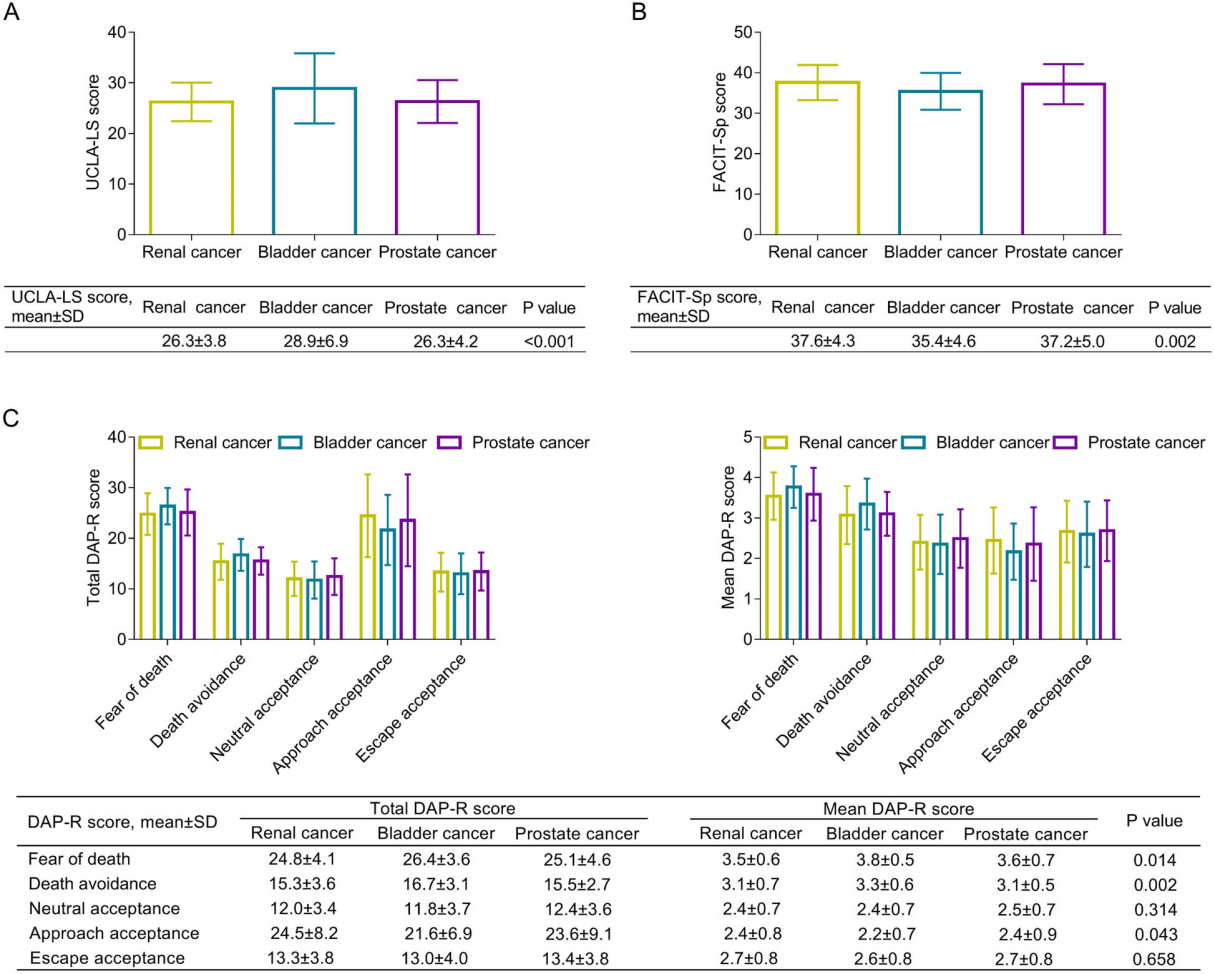
Comparison of University of California and Los Angeles loneliness scale (UCLA-LS) score (**A**), functional assessment of chronic illness therapy-spiritual well-being (FACIT-Sp) score (**B**), as well as total and mean death attitude profile-revised (DAP-R) score (**C**) among renal, bladder, and prostate cancer patients. Data are reported as means±SD (*t*-test).

UCLA-LS score was different among bladder cancer, prostate cancer, and renal cancer patients, which was highest in bladder cancer patients and relatively low in prostate and renal cancer patients (P<0.001) (Figure 2A). FACIT-Sp score also differed among bladder, prostate, and renal cancer patients, which was lowest in bladder cancer patients and relatively high in prostate and renal cancer patients (P=0.002) (Figure 2B). In terms of total DAP-R score, fear of death (P=0.014) and death avoidance (P=0.002) were highest in bladder cancer patients and relatively low in prostate and renal cancer patients. Approaching death acceptance exhibited an opposite trend: lowest in bladder cancer patients and relatively high in prostate and renal cancer patients (P=0.043). Neutral acceptance and escape acceptance did not differ among the three types of patients (both P>0.05). The same findings were found when the mean DAP-R score was compared among renal, bladder, and prostate cancer patients (Figure 2C).

### Independent factors related to UCLA-LS score in urological cancer patients

Univariate linear regression analysis revealed that gender (female) (B=1.764, P=0.013), marital status (single/divorced/widowed) (B=1.927, P=0.003), bladder cancer (*vs* prostate and renal cancer) (B=2.604, P<0.001), pathological grade at diagnosis (B=1.089, P=0.017), and systemic treatment (yes) (B=1.823, P=0.002) were related to a higher UCLA-LS score. Higher levels of education (B=-1.036, P=0.021) and prostate cancer (*vs* bladder and renal cancer) (B=-1.431, P=0.019) were associated with a lower UCLA-LS score. Further backward multivariate linear regression analyses suggested that marital status (single/divorced/widowed) (B=1.460, P=0.023), bladder cancer (*vs* prostate and renal cancer) (B=1.610, P=0.014), and systemic treatment (yes) (B=1.425, P=0.020) were independently correlated with a higher UCLA-LS score (Table 2).

**Table 2:** Linear regression analysis for UCLA-LS score in urological cancer patients.

Items	B	SE	Standardized B	*t* value	P value
Univariate linear regression model					
Age	0.030	0.030	0.054	0.970	0.333
Gender (female)	1.764	0.706	0.138	2.499	0.013
Smoking history (yes)	-0.514	0.609	-0.047	-0.844	0.399
Drinking history (yes)	0.389	0.640	0.034	0.608	0.544
Marital status (single/divorced/widowed)	1.927	0.644	0.164	2.991	0.003
Employment status (Unemployed)	0.902	0.603	0.083	1.495	0.136
Level of education	-1.036	0.447	-0.128	-2.320	0.021
Location (rural)	-0.363	0.828	-0.024	-0.438	0.661
Hypertension (yes)	0.628	0.605	0.058	1.039	0.300
Hyperlipidemia (yes)	0.172	0.703	0.014	0.244	0.807
Diabetes (yes)	1.063	0.800	0.074	1.328	0.185
Renal cancer (*vs* bladder and prostate cancer)	-1.295	0.674	-0.106	-1.922	0.055
Bladder cancer (*vs* prostate and renal cancer)	2.604	0.611	0.231	4.264	<0.001
Prostate cancer (*vs* bladder and renal cancer)	-1.431	0.609	-0.130	-2.348	0.019
Pathological grade at diagnosis	1.089	0.453	0.133	2.406	0.017
TNM stage at diagnosis	0.578	0.327	0.098	1.767	0.078
Surgery treatment (yes)	0.425	1.215	0.019	0.350	0.727
Systemic treatment (yes)	1.823	0.590	0.170	3.091	0.002
Local treatment (yes)	0.594	0.602	0.055	0.987	0.324
Other treatments (yes)	-0.067	0.659	-0.006	-0.101	0.920
Backward multivariate linear regression model					
Gender (female)	1.306	0.731	0.102	1.787	0.075
Marital status (single/divorced/widowed)	1.460	0.639	0.125	2.285	0.023
Bladder cancer (*vs* prostate and renal cancer)	1.610	0.649	0.143	2.482	0.014
Pathological grade at diagnosis	0.764	0.445	0.093	1.717	0.087
Systemic treatment (yes)	1.425	0.608	0.133	2.343	0.020
Local treatment (yes)	1.004	0.605	0.093	1.661	0.098

UCLA-LS: University of California and Los Angeles loneliness scale; SE: standard error; TNM: tumor-node-metastasis.

### Independent factors related to FACIT-Sp score in urological cancer patients

Univariate linear regression analysis showed that marital status (single/divorced/widowed) (B=-1.377, P=0.016), bladder cancer (*vs* prostate and renal cancer) (B=-1.948, P<0.001), pathological grade at diagnosis (B=-0.885, P=0.028), and systemic treatment (yes) (B=-1.744, P=0.001) were related to a lower FACIT-Sp score. Renal cancer (*vs* bladder and prostate cancer) was associated with a higher FACIT-Sp score (B=1.248, P=0.036). Further backward multivariate linear regression analyses disclosed that marital status (single/divorced/widowed) (B=-1.363, P=0.020), bladder cancer (*vs* prostate and renal cancer) (B=-1.913, P=0.002), pathological grade at diagnosis (B=-0.855, P=0.037), surgery treatment (yes) (B=-3.139, P=0.005), systemic treatment (yes) (B=-2.323, P<0.001), and local treatment (yes) (B=-1.560, P=0.020) were independently associated with a lower FACIT-Sp score (Table 3).

**Table 3:** Linear regression analysis for FACIT-Sp score in urological cancer patients.

Items	B	SE	Standardized B	*t* value	P value
Univariate linear regression model					
Age	-0.005	0.027	-0.010	-0.186	0.852
Gender (female)	-0.946	0.626	-0.084	-1.511	0.132
Smoke history (yes)	0.249	0.537	0.026	0.464	0.643
Drink history (yes)	-0.075	0.565	-0.007	-0.133	0.895
Marital status (single/divorced/widowed)	-1.377	0.571	-0.133	-2.411	0.016
Employment status (unemployed)	-0.397	0.534	-0.041	-0.744	0.457
Level of education	0.766	0.395	0.107	1.939	0.053
Location (rural)	-0.232	0.730	-0.018	-0.317	0.751
Hypertension (yes)	-0.539	0.533	-0.056	-1.010	0.313
Hyperlipidemia (yes)	0.238	0.620	0.021	0.384	0.702
Diabetes (yes)	-0.989	0.706	-0.078	-1.401	0.162
Renal cancer (*vs* bladder and prostate cancer)	1.248	0.593	0.116	2.104	0.036
Bladder cancer (*vs* prostate and renal cancer)	-1.948	0.543	-0.196	-3.589	<0.001
Prostate cancer (*vs* bladder and renal cancer)	0.840	0.540	0.086	1.556	0.121
Pathological grade at diagnosis	-0.885	0.400	-0.122	-2.214	0.028
TNM stage at diagnosis	-0.540	0.288	-0.104	-1.872	0.062
Surgery treatment (yes)	-1.161	1.070	-0.060	-1.086	0.278
Systemic treatment (yes)	-1.744	0.519	-0.184	-3.361	0.001
Local treatment (yes)	-0.559	0.530	-0.059	-1.054	0.293
Other treatments (yes)	-0.742	0.580	-0.071	-1.280	0.201
Backward multivariate linear regression model					
Age	0.056	0.029	0.116	1.939	0.053
Marital status (single/divorced/widowed)	-1.363	0.585	-0.132	-2.331	0.020
Diabetes (yes)	-1.208	0.688	-0.095	-1.755	0.080
Renal cancer (*vs* bladder and prostate cancer)	-1.661	0.876	-0.155	-1.895	0.059
Bladder cancer (*vs* prostate and renal cancer)	-1.913	0.626	-0.193	-3.058	0.002
Pathological grade at diagnosis	-0.855	0.408	-0.118	-2.098	0.037
Surgery treatment (yes)	-3.139	1.122	-0.163	-2.797	0.005
Systemic treatment (yes)	-2.323	0.649	-0.245	-3.579	<0.001
Local treatment (yes)	-1.560	0.669	-0.164	-2.331	0.020

FACIT-Sp: Functional assessment of chronic illness therapy-spiritual well-being; SE: standard error; TNM: tumor-node-metastasis.

### Independent factors related to DAP-R score in urological cancer patients

Marital status (single/divorced/widowed) (B=1.371, P=0.009), bladder cancer (*vs* prostate and renal cancer) (B=1.092, P=0.029), and surgery treatment (yes) (B=1.021, P=0.026) were related to a higher fear of death evaluated by DAP-R score. Bladder cancer (*vs* prostate and renal cancer) (B=1.101, P=0.003) and surgery treatment (yes) (B=0.824, P=0.019) were correlated with a higher death avoidance evaluated by DAP-R score. Gender (female) (B=-1.248, P=0.008) was related to a lower neutral acceptance evaluated by DAP-R score. Bladder cancer (*vs* prostate and renal cancer) (B=-2.063, P=0.030) and pathological grade at diagnosis (B=-1.726, P=0.013) were associated with a lower approaching death acceptance evaluated by DAP-R score. Renal cancer (*vs* bladder and prostate cancer) (B=1.500, P=0.027) and local treatment (yes) (B=1.838, P=0.001) were associated to a higher escape acceptance evaluated by DAP-R score (Supplementary Table S1).

### Correlation of UCLA-LS, FACIT-Sp, and DAP-R scores in urological cancer patients

UCLA-LS score was negatively related to the FACIT-Sp score (r=-0.468, P<0.001), as well as neutral acceptance (r=-0.384, P<0.001) and approaching death acceptance (r=-0.319, P<0.001) evaluated by the DAP-R score. However, the UCLA-LS score was positively associated with fear of death (r=0.350, P<0.001) and death avoidance (r=0.402, P<0.001) evaluated by the DAP-R score in urological cancer patients. FACIT-Sp score was negatively linked to fear of death (r=-0.386, P<0.001) and death avoidance (r=-0.334, P<0.001) evaluated by the DAP-R score in urological cancer patients. FACIT-Sp score was positively related to neutral acceptance (r=0.325, P<0.001) and approaching death acceptance (r=0.331, P<0.001) evaluated by the DAP-R score in urological cancer patients. Notably, although statistical significance was found between UCLA-LS score and escape acceptance (r=0.292, P<0.001) evaluated by DAP-R score, as well as between FACIT-Sp score and escape acceptance (r=-0.275, P<0.001) evaluated by DAP-R score, the correlation coefficient was relatively small (Table 4).

**Table 4:** Correlations of UCLA-LS, FACIT-Sp, and DAP-R in urological cancer patients.

Items	UCLA-LS		FACIT-Sp	
	r value	P value	r value	P value
FACIT-Sp	-0.468	<0.001	-	-
DAP-R				
Fear of death	0.350	<0.001	-0.386	<0.001
Death avoidance	0.402	<0.001	-0.334	<0.001
Neutral acceptance	-0.384	<0.001	0.325	<0.001
Approach acceptance	-0.319	<0.001	0.331	<0.001
Escape acceptance	0.292	<0.001	-0.275	<0.001

UCLA-LS: University of California and Los Angeles loneliness scale; FACIT-Sp: Functional assessment of chronic illness therapy-spiritual well-being; DAP-R: Death attitude profile-revised.

## Discussion

Urological cancer patients usually suffer from social or self-isolation, poor spiritual status, and fear of death, which might result in a reduced quality of life and increased mortality (14,19). The present study found that loneliness was higher and spiritual well-being was lower in urological cancer patients than in healthy controls, which was partly in line with previous studies that indicated that loneliness and reduced spiritual well-being were prevalent in cancer patients (12,14,20). Urological cancer patients have low self-esteem and might also feel a loss of self-worth, which results in less social interaction with others and ultimately contributes to increased loneliness (21,22); it should be mentioned that a previous study reported that loneliness was indirectly responsible for the occurrence of depression in prostate cancer patients (23). Considering that loneliness is elevated in urological cancer patients, more attention should be given to these patients, and the improvement of psychological management is warranted. In addition, urological cancer patients bear huge financial stress and worry about their physical functions and tumor advancement, which impairs their spiritual well-being (24-26).

Regarding death perception, the current study showed that fear of death, death avoidance, and approaching death acceptance were higher but neutral acceptance of death was lower in urological cancer patients than in healthy controls. It is known that urological cancer patients experience tremendous physical pain and psychological stress, which might make them feel that death is close (20,27); therefore, fear of death and death avoidance are obvious in urological cancer patients. In addition, the survival of urological cancer patients is shorter than normal people (2); hence, urological cancer patients might consider that life is unfair to them, leading to lower neutral acceptance of death. Moreover, urological cancer patients face the fact that death is inevitable, and the life they experience is tough and miserable; thus, seeking hope and thinking about death positively is important for them (10,28). This might lead to a higher death acceptance.

Notably, loneliness, spiritual well-being, and death perception can further affect the clinical outcomes of cancer patients. For example, a previous study reported that a decreased spiritual well-being was correlated with impaired quality of life and emotional distress in cancer patients (14). However, the current research was a case-control study, which did not include follow-up data; thus, further studies should be conducted to explore this aspect in urological cancer patients.

The present study further observed that increased loneliness and reduced spiritual well-being were obvious in bladder cancer patients. The potential reasons for this might be that: 1) the symptom of hematuria occur in the early stage of bladder cancer compared to prostate and renal cancer, which would make bladder cancer patients feel fear and shame and further contribute to reduced interaction with others, leading to feelings of loneliness (21,29); 2) bladder cancer patients suffer from huge mental stress, and some of them would even need to carry a urine pouch for the rest of their life, which causes great inconvenience; however, prostate cancer and renal cancer patients do not need to carry a urine pouch (24,30-32), which might explain the higher spiritual well-being in prostate and renal cancer patients. This finding was further confirmed by backward multivariate linear regression analysis. Death perception, fear of death, and death avoidance were elevated but approaching death acceptance was lower in bladder cancer patients (*vs* renal and prostate cancer). The explanations could be that: 1) the survival of bladder cancer patients is relatively short (33); meanwhile, these patients develop hematuria at an early stage (29); in addition, the pain from this disease and its treatments make them feel that death is close and terrible; hence, fear of death and death avoidance are increased (24,30); 2) loss of physical functions and the psychological burden contribute to despair in bladder cancer patients, which results in decreased approaching death acceptance in these patients (30,34).

The present study also found that single/divorced/widowed status and systemic treatment were independently associated with increased loneliness in urological cancer patients. The potential reasons would be that: 1) single/divorced/widowed patients are more likely to lack social interaction, resulting in increased loneliness (35); 2) systemic treatment causes great physical discomfort to the patients, as well as huge financial stress to the family; these factors affect interaction with society and family, leading to increased loneliness (36). Concurrently, single/divorced/widowed status and higher pathological grade at diagnosis, as well as surgery, systemic, and local treatments were independently associated with decreased spiritual well-being in urological cancer patients. The reasons behind this might be that: 1) as discussed above, single/divorced/widowed patients are more likely to feel unsupported, which affects their social well-being (35); 2) a higher pathological grade causes a great fear of disease in urological patients (37); 3) due to the specificity of the urological tumor sites, urinary and sexual functions are negatively affected and patients might also worry about the recurrence of cancer after surgery, local, and systemic treatments (38,39).

Limitations should be mentioned in this study. Firstly, this was a single-center study; therefore, selection bias might exist. Secondly, this study was conducted in China; thus, the findings of this study cannot be generalized due to the different sociodemographic and cultural characteristics of urological cancer patients in other regions. Thirdly, the UCLA-LS, FACIT-Sp, and DAP-R scores were self-assessed, which might lead to assessment bias. Fourthly, multiple-time assessments of loneliness, spiritual well-being, and death perception were not carried out in this study, and this could be a direction for further studies. Fifthly, considering that the number of caregivers and the relationship between caregivers and urological cancer patients can potentially affect the loneliness, spiritual well-being, and death perception of patients, further studies could explore the role of caregivers in these statuses.

In conclusion, physicians should pay close attention to patients with the above mentioned risk factors, since these risk factors may affect the status of loneliness, spiritual well-being, and death perception in urological cancer patients and further influence the quality of life and prognosis of these patients. Therefore, our findings may provide a reference to improve the psychological management of urological cancer patients.

## Supplementary Material

Click here to view [pdf].
